# Spontaneous Hepatic Subcapsular Haematoma Due to Hepatic Artery Pseudo-Aneurysm Rupture: A Rare Case in an Elderly Male

**DOI:** 10.7759/cureus.97865

**Published:** 2025-11-26

**Authors:** Hameed Ur Raheem, Aamina Ateeque, Hamza Shahzad, Zeeshan Shafqat, Aroob Zahra, Rimsha Rashid

**Affiliations:** 1 Medicine and Surgery, Gloucestershire Royal Hospital, Gloucester, GBR; 2 Medicine and Surgery, University Hospital Crosshouse, Kilmarnock, GBR; 3 Public Health, University of Dundee, Dundee, GBR; 4 Medicine, Doncaster Royal Infirmary, Doncaster, GBR; 5 Oral and Maxillofacial Surgery, University Hospitals of Leicester, Leicester, GBR; 6 Emergency Medicine, Queen Elizabeth University Hospital, Glasgow, GBR

**Keywords:** hepatic bleeding, idiopathic pseudoaneurysm, interventional radiology-guided embolization, right hepatic artery pseudoaneurysm, subcapsular hepatic hematoma

## Abstract

Hepatic artery pseudo-aneurysm (HAPA) is a rare vascular complication that is typically associated with trauma or hepatobiliary procedures. Spontaneous rupture usually occurs without preceding trauma or intervention and is exceptionally uncommon. This case highlights a rare presentation of HAPA leading to a hepatic subcapsular haematoma in an elderly male. An 80-year-old male was admitted with confusion and had an unwitnessed fall at home. Initial investigations demonstrated severe hyponatraemia, elevated C-reactive protein, and left-basal consolidation on chest X-ray, along with a subsequent diagnosis of syndrome of inappropriate ADH secretion (SIADH) due to pneumonia. During admission, the patient then developed hypotension and a significant drop in haemoglobin. Subsequent contrast-enhanced CT scan revealed a hepatic subcapsular haematoma caused by a ruptured HAPA. The patient was urgently referred to interventional radiology, where selective catheterisation and coil embolisation were performed to secure complete haemostasis. Post-procedure, the patient’s haemoglobin was stabilised, and liver function improved. This case illustrates that spontaneous rupture of a HAPA should be considered in elderly or septic patients with unexplained anaemia and abnormal liver function. Early imaging with contrast-enhanced CT scan and timely interventional radiology are essential for successful management.

## Introduction

Hepatic artery pseudo-aneurysm (HAPA) is a rare complication following various hepatobiliary and surgical interventions, including percutaneous cholangiography, biliary stent placement, laparoscopic and open cholecystectomy, biliary reconstruction and Whipple’s procedure [1]. If rupture occurs, it can lead to life-threatening haemorrhage and formation of a subcapsular haematoma. The estimated incidence of HAPA is 0.002% [2].

Spontaneous rupture of a pseudo-artery aneurysm occurring without any preceding trauma is very rare. We present a rare case of subcapsular liver haematoma accompanied by a hepatic artery pseudo-aneurysm, occurring in the absence of any noticeable liver injury, existing liver disorders, blood coagulation issues, or previous interventions involving the hepatobiliary system.

## Case presentation

An 80-year-old male was admitted to Gloucestershire Royal Hospital on 5 May 2025, through the emergency department, with increasing confusion and an unwitnessed fall at home. According to family members, he had been progressively disoriented, and his condition worsened overnight. On arrival, initial observations showed a heart rate of 94 bpm, blood pressure 103/59 mmHg, temperature 36.9 °C, and oxygen saturation 98% on room air. He was alert but confused with a Glasgow Coma Scale (GCS) of 14/15, mild tachycardia, a pansystolic murmur loudest at the mitral area and mild generalised abdominal tenderness. His past medical history included eye cataract surgery, bilateral Dupuytren's contracture repair and psoriasis, and asthma. His medication history includes cholecalciferol, salbutamol inhaler, clobetasol ointment and emollient.

Further investigations demonstrated severe hyponatraemia, elevated C-reactive protein and left basal consolidation on chest X-ray. CT spine and CT head demonstrated no acute pathology. The viral swab and urine cultures returned negative results. Paired serum and urine osmolality confirmed syndrome of inappropriate antidiuretic hormone secretion (SIADH), most likely due to pneumonia. Therefore, the patient was commenced on IV piperacillin/tazocin and 1L/24-hour fluid restriction. Furthermore, during the admission, he also developed diarrhoea and symptomatic hypotension with systolic blood pressure readings between 79 and 89 mmHg. Repeat blood test results showed a drop in haemoglobin from 144 g/l to 97 g/l, with deranged liver enzymes and several documented episodes of melena. Subsequently, an abdominal ultrasound done on 15/5/25 demonstrated two focal hepatic lesions initially thought to represent abscesses, prompting gastroenterology consultation.

**Figure 1:**
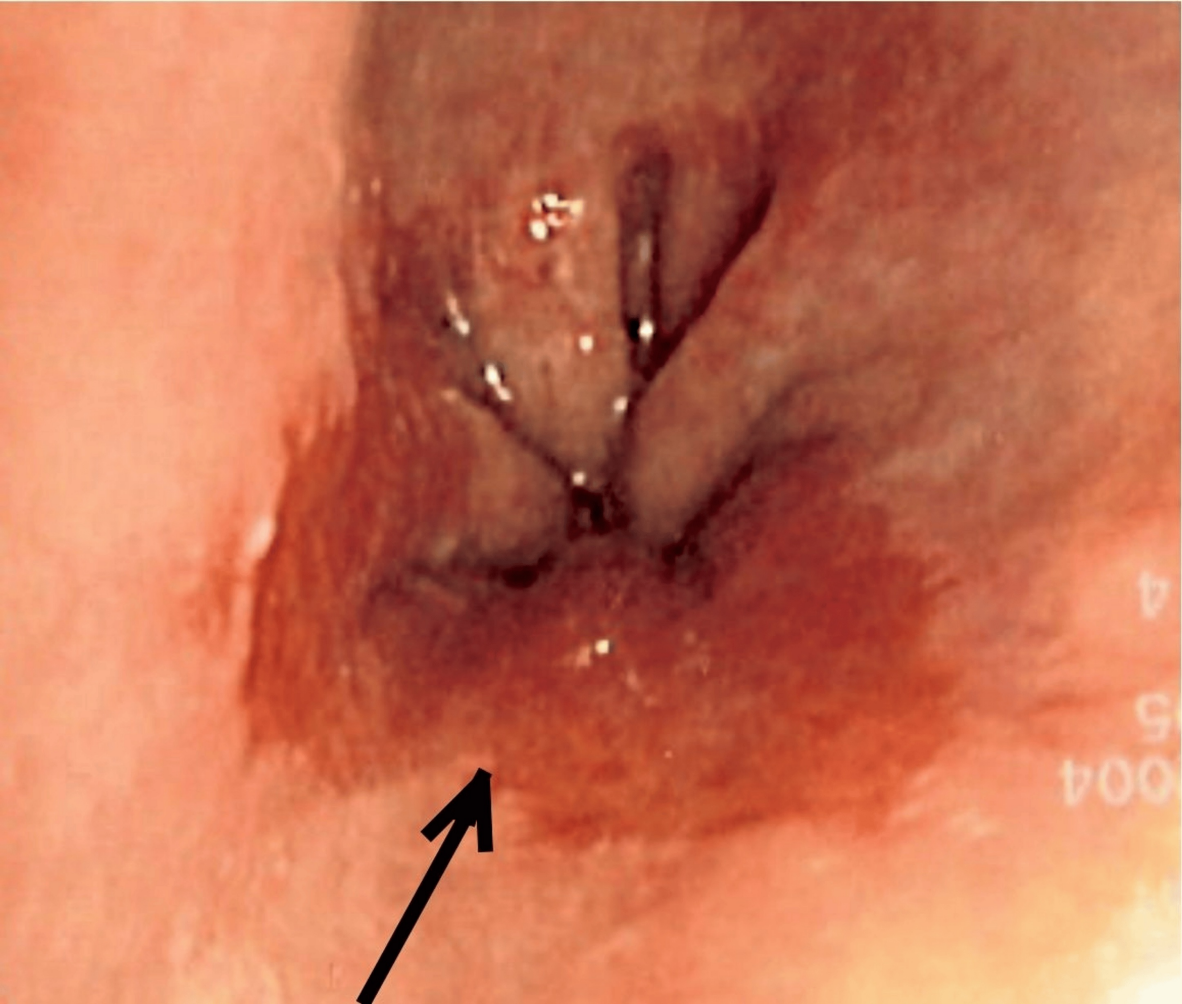
Endoscopic view showing Barrett’s oesophagus

An upper gastrointestinal endoscopy revealed incidental Barrett’s oesophagus, but no evidence of active upper gastrointestinal bleed as shown in Figure 1.

**Figure 2:**
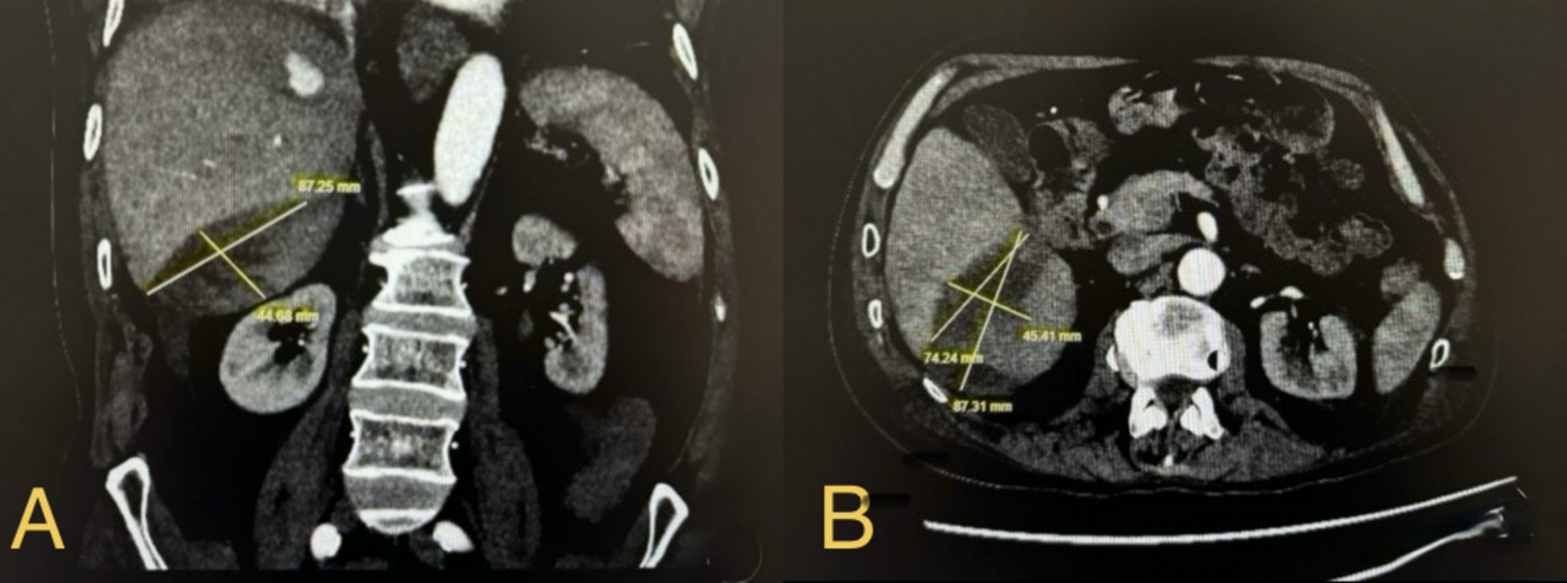
Contrast-enhanced CT scan of the abdomen showing a large right hepatic subcapsular haematoma: (A) coronal and (B) axial views

Due to the continuous decline in haemoglobin, worsening liver function tests and previous ultrasound findings, a contrast-enhanced CT scan of the abdomen was performed on 17 May 2025, which demonstrated a large right hepatic subcapsular haematoma with active contrast extravasation arising from a pseudo-aneurysm in hepatic segments VII-VIII on the arterial phase of the scan, as shown in Figure 2.

**Figure 3:**
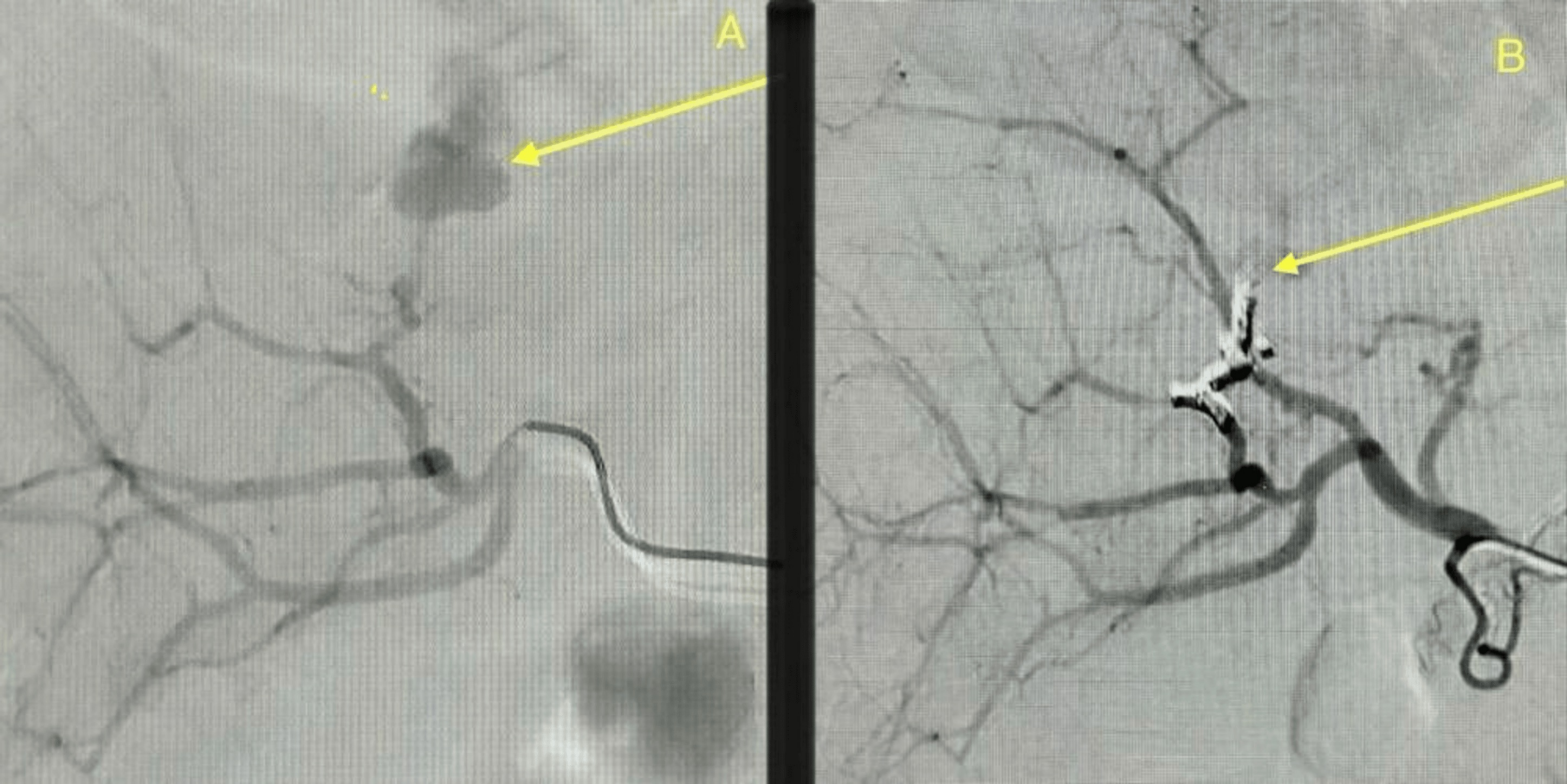
Hepatic artery pseudo-aneurysm and post-embolisation outcome (A) Digital subtraction angiography demonstrates a large pseudo-aneurysm arising from the segment VII branch of the hepatic artery. (B) Follow-up angiogram following coil embolisation using multiple Concerto coils shows complete exclusion of the pseudo-aneurysm with no residual contrast filling, confirming the technical success of the procedure.

The patient was urgently referred to interventional radiology, where angiography confirmed a sizeable aneurysm with active arterial filling. On 19 May 2025, through the right common femoral access, selective catheterisation of the feeding branch was achieved, and coiled embolisation was performed using multiple micro coils, resulting in complete angiographic occlusion, as shown in Figure 3.

Following the procedure, the patient became hemodynamically stable with no further evidence of bleeding. Post-procedure, his haemoglobin remained stable, and the liver function test gradually improved. He tolerated diet advancements, had an iron transfusion prior to discharge and recovered well.

The patient was discharged on 6 June 2025, in a stable condition with outpatient follow-up and a planned triphasic CT scan to confirm resolution of the haematoma. His follow-up triphasic CT scan of the liver showed evidence of successful embolisation of the segment VII hepatic pseudo-aneurysm with no definitive residual filling defect seen, and a large subcapsular hepatic haematoma evolved into a well-matured, contained collection.

## Discussion

HAPA is an uncommon vascular lesion that forms after the disruption of the arterial wall, creating a false lumen contained by surrounding tissue [3]. Unlike a true aneurysm, it lacks all three layers of the arterial wall, making it more prone to rupture. It usually follows trauma or hepatobiliary procedures but rarely occurs spontaneously [4]. This patient had no history of trauma, hepatobiliary procedure or liver pathology. The pseudo-aneurysm likely developed secondary to infection-related endothelial injury and transient hypotension, which may have weakened the arterial wall and predisposed it to rupture. The subsequent arterial bleed accumulated beneath the hepatic capsule, producing a large subcapsular haematoma.

The clinical presentation of HAPA can be subtle, ranging from right upper quadrant pain and melena to hypovolemic shock. In many cases, the diagnosis is delayed, as the laboratory findings are non-specific. In this case, the first indication of bleeding was a rapid decline in haemoglobin level, melena and deranged liver enzymes, prompting imaging that revealed the underlying pathology.

Contrast-enhanced CT is regarded as the method of choice for evaluating suspected hepatic haemorrhage, as it can detect both the haematoma and the location of any active bleeding. After a diagnosis is made, selective angiography accompanied by coil embolisation is still considered the standard treatment, ensuring definitive control of bleeding while maintaining liver tissue. Surgical procedures are now only considered in situations where embolisation does not succeed or if the patient continues to be unstable.

There are only a few instances of spontaneous rupture of HAPAs reported in medical literature. Joh et al. (2021) and Al Tamimi et al. (2019) discussed similar cases involving a hepatic subcapsular haematoma caused by the rupture of a pseudo-aneurysm without any prior trauma or medical interventions [2,5]. In contrast, our patient was experiencing both an infection and hypotension, which may have increased the fragility of the arterial walls.

This case emphasises that hepatic artery pseudo-aneurysm rupture should be considered in elderly or septic patients who present with unexplained anaemia and abnormal liver function, regardless of the presence of clear risk factors. Timely imaging and interventional radiology are crucial and should be prioritised when the clinical situation is ambiguous.

## Conclusions

Spontaneous rupture of a hepatic artery pseudo-aneurysm is rare and typically occurs after trauma or hepatobiliary procedures. As is evident from the literature available, it can also occur without any trauma or intervention. In this case, infection-related endothelial injury and hypotension may have contributed to the rupture.

Early diagnosis through contrast-enhanced CT scan and coil embolisation is the gold standard management option, as supported by several studies. This case highlights the importance of considering HAPA rupture in elderly or septic patients presenting with unexplained anaemia and abnormal liver function, with early imaging and intervention being key to preventing fatal outcomes.
